# Systemic tuberculosis by *MYCOBACTERIUM BOVIS* in a free-ranging MARSICAN brown bear (*URSUS ARCTOS MARSICANUS*): a Case report

**DOI:** 10.1186/s12917-019-1910-0

**Published:** 2019-05-17

**Authors:** Rosario Fico, Alessia Mariacher, Alessia Franco, Claudia Eleni, Erika Ciarrocca, Maria Lodovica Pacciarini, Antonio Battisti

**Affiliations:** 1 Istituto Zooprofilattico Sperimentale delle Regioni Lazio e Toscana, Viale Europa 30, 58100 Grosseto, Italy; 2 Istituto Zooprofilattico Sperimentale delle Regioni Lazio e Toscana, Via Appia Nuova 1411, 00178 Rome, Italy; 3 Istituto Zooprofilattico Sperimentale della Lombardia e dell’Emilia-Romagna, National Reference Laboratory for Bovine Tuberculosis, Via A. Bianchi 9, 25124 Brescia, Italy

**Keywords:** Bovine tuberculosis, Marsican brown bear, *Mycobacterium bovis*

## Abstract

**Background:**

*Mycobacterium bovis* is known to have a wide host range and has been isolated from numerous free-ranging wildlife species, carnivores included. In bears, *M. bovis* has been previously reported only from a culture of pooled lymph nodes of a black bear (*Ursus americanus*) in the absence of lesions. The aims of this study were to describe gross and microscopic pathological findings of *M. bovis* tuberculosis in a deceased Marsican brown bear (*Ursus arctos marsicanus*).

**Case presentation:**

In March 2014, an adult female Marsican brown bear was found in the Abruzzo, Lazio and Molise National Park (Italy) showing severe non-specific clinical signs. The animal died soon after its discovery and the carcass was submitted to post-mortem examination to identify the cause of death. The bear was diagnosed with a severe *Mycobacterium bovis* infection, with both pathological and microbiological aspects suggesting ongoing generalization. A presumptive diagnosis of mycobacterial infection was initially made based on gross findings. Histopathology showed the presence of acid-fast bacilli in all sampled tissues along with poorly organized granulomatous lesions. Slow-growing *Mycobacterium* sp. was isolated from multiple organs (intestine, mesenteric lymph nodes, liver, spleen, lung and kidneys). The PCR and sequencing algorithm identified the *Mycobacterium* sp. isolate as *M. bovis*. Spoligotyping demonstrated that the *M. bovis* isolate belonged to spoligotype SB0120.

**Conclusions:**

This is the first report of lethal *M. bovis* tuberculosis infection in a free-ranging brown bear. This pathogen could have serious adverse effects in an endangered relic population such as the Marsican brown bear. Stricter application of health regulations in force, surveillance of *M. bovis* infections in wild ungulates and carnivore scavengers, along with dismissal of supplementary feeding points intended for cattle or wildlife, are warranted to control the presence of bovine tuberculosis in wild and domestic animals in protected areas.

## Background

*Mycobacterium bovis*, causative agent of bovine tuberculosis, is known to have a wide host range and is often maintained in complex transmission cycles at the interface where wildlife and livestock meet [1]. *M. bovis* has been previously isolated from numerous free-ranging non-cervid wildlife species, carnivores included [2–9]. Nonetheless, in bears, *M. bovis* was only cultured from pooled lymph nodes of a black bear (*Ursus americanus*) in the absence of gross or histological lesions [2].

The aims of this study were to describe gross and microscopic pathological findings of *M. bovis* tuberculosis in a deceased Marsican brown bear (*Ursus arctos marsicanus*). The report also aims to shed light on possible source of infection, providing identification and molecular characterization of the etiological agent.

## Case presentation

In March 2014, an adult female Marsican brown bear died in the Province of L’Aquila, in the territory of the Abruzzo, Lazio and Molise National Park (Italy). The animal was found alive and showing severe, non-specific clinical signs (dyspnoea, hyper salivation and disorientation) and died soon after its discovery by park rangers. The carcass was submitted by local authorities for forensic post-mortem examination to ascertain the cause of death and to rule out any illegal activities.

**Fig. 1:**
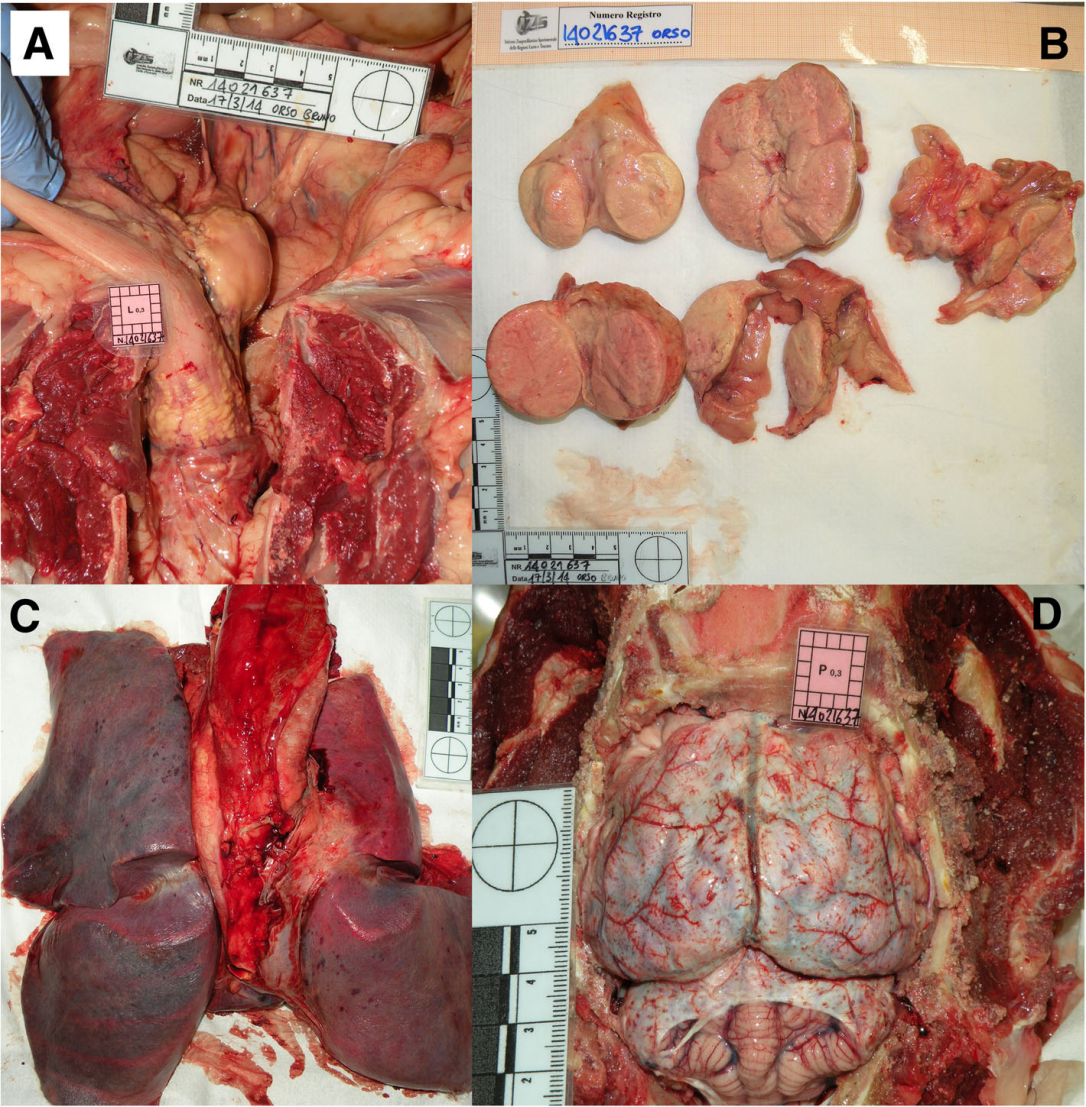
Marsican brown bear, necroscopic examination. **a**) Necrotic plaques on the external surface of the rectum wall, extending to the adjacent lymph node; **b**) Cut surfaces of enlarged mesenteric lymph nodes, showing extensive necrosis; **c**) Pulmonary oedema and multifocal subpleural petechiae; **d**) Meningeal hyperaemia

A forensic necroscopic exam was performed including complete skinning of the carcass, skull opening and photographic documentation with metric reference. The bear was in fair body condition. Pale mucous membranes were observed at external examination. After skinning, a fair amount of subcutaneous adipose tissue was observed, adequate to the season. At the opening of abdominal cavity, mild peritonitis and peritoneal serohemorrhagic effusion were observed. Small and large intestines were markedly thickened and on the external surface of rectum wall, coalescing necrotic plaques were seen, extending to an adjacent lymph node (Fig. 1, a). The stomach and intestines were empty. A diffuse mild catarrhal gastritis was observed in the fundic region. All mesenteric lymph nodes were massively enlarged and their normal anatomy at cut surfaces was effaced by extensive necrosis (Fig. 1, b). The liver was enlarged and the spleen was pale and enlarged. Three placental scars were observed in the uterus. Gross findings of the upper airways included greenish rhinopharyngeal catarrhal exudate and laryngeal oedema and congestion. In the thoracic cavity, pulmonary oedema and subpleural petechiae were observed (Fig. 1, c). After skull opening, meningeal hyperaemia was observed (Fig. 1, d). A presumptive diagnosis of systemic tuberculosis was initially made based on gross findings, and later confirmed by histology and microbiology.

**Fig. 2:**
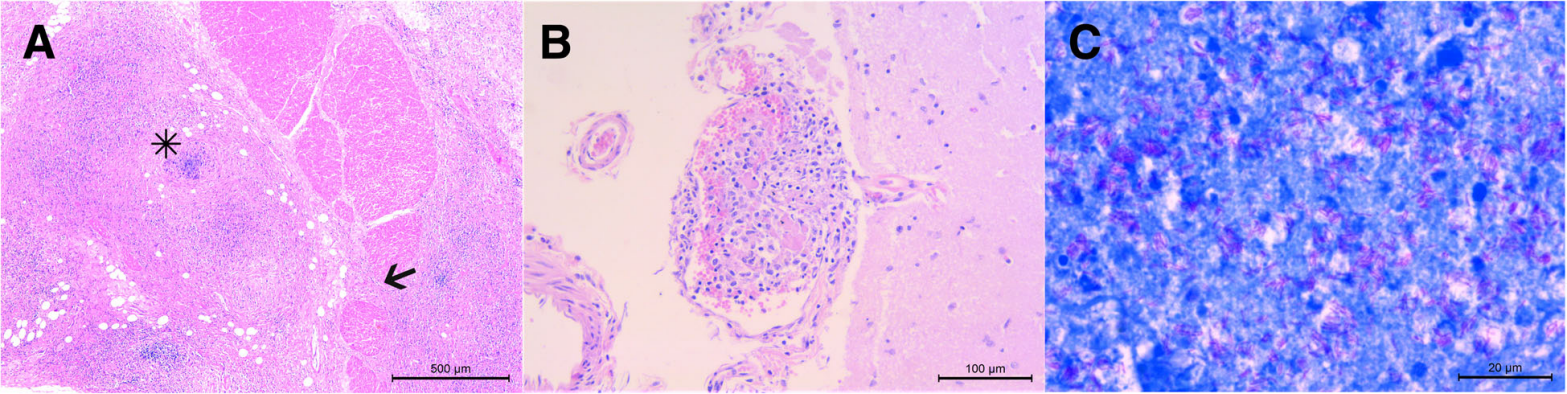
Histopathology. **a**) Rectum - massive necrosis of the intestinal wall, with infiltration (arrow) and multifocal severe extension in the peritoneal serosa (asterisk) (hematoxylin and eosin, magnification 5x, scale bar = 500 μm); **b**) Meninges - meningeal granuloma (hematoxylin and eosin, magnification 20x, scale bar = 100 μm); **c**) Lymph nodes - acid-fast bacilli, brilliant pink in color, both in macrophages and extracellularly (Ziehl-Neelsen, magnification 100x, scale bar = 20 μm)

Samples were collected from all the major organs and from all the tissues affected by gross lesions. Tissue samples for histopathology were formalin-fixed, embedded in paraffin wax, sectioned at 4 μm and stained with HE and Ziehl-Neelsen. Histologically, massive transmural necrosis was observed in the intestine wall with multifocal extension in the peritoneal serosa (Fig. 2, a). Multiple granulomatous foci suggestive of tuberculosis, showing poor organization and composed of lymphocytes, plasma cells, macrophages and few neutrophils, were found mixed with necrotic debris. Similar lesions were also present in the mesenteric lymph nodes. In liver, kidneys and brain, granulomatous foci were respectively seen in the periportal area, in the interstitium and in the meninges (Fig. 2, b). A mild multifocal perisplenitis was also detected. Acid-fast bacilli were observed within tubercles, both in macrophages and extracellularly (Fig. 2, c) in all the examined organs (skin, lymph nodes, liver, spleen, intestines, peritoneum, kidneys, lung, myocardium and brain).

Samples from multiple organs and fluids (liver, spleen, pooled lymph nodes, intestines, kidneys, lung, brain, intracardial blood clot and peritoneal effusion) were submitted to cultures for bacterial pathogens, including Mycobacteria. Cultures for bacterial pathogens were performed on blood agar incubated at 37 °C in aerobic, capnophilic (10% CO_2_ atmosphere) and anaerobic conditions following standard procedures. Cultures for *Mycobacterium* spp. were performed on enrichment liquid (Middlebrook 7H9) and solid (Coletsos + ossein) media, both added with pyruvate, following international recommendations [10], with an incubation at 37 °C in aerobic conditions for up to 12 weeks. Identification at species level of *Mycobacterium* spp. was obtained by an algorithm including a multiplex PCR approach for *Mycobacterium* genus, *M. tuberculosis-complex* [11] and sequencing the gyrB gene [12]. Spoligotyping was performed as previously described [13]. Slow-growing *Mycobacterium* sp. was isolated from multiple organs (intestine, mesenteric lymph nodes, liver, spleen, lung and kidneys). The PCR and sequencing algorithm identified the *Mycobacterium* sp. isolate at species level as *M. bovis*. Spoligotyping demonstrated that the *M. bovis* isolate belonged to spoligotype SB0120. Since this is the dominant profile in the Italian cattle population [14], for further molecular characterization and for purposes of molecular epidemiology, a Variable Tandem Repeat approach with a “core” group 12 loci as previously described [14]. The allelic pattern obtained was the following: ETR-A: 5, ETR-B: 5; ETR-C: 5; ETR-D: 3; ETR-E: 3, MIRU26: 5; QUB11A: 10, QUB11B: 4; QUB15: 3; QUB1895: 4, QUB26: 4; QUB3232: 6.

This pattern was identical to isolates from an outbreak in semi-free-ranging bovine animals, which had been first detected in June 2012 in the same municipality.

*Staphylococcus schleiferi* subsp. *coagulans* was isolated from the intracardial clot and peritoneal fluid.

Liver samples were submitted to toxicological analysis (Gas Chromatography - Mass Spectrometry, GC-MS) for organophosphate, carbamate, and organochlorine pesticides with negative results.

## Discussion and conclusions

The Marsican brown bear is classified as a critically endangered species by the International Union for Conservation of Nature IUCN [15]. The estimated size of this isolated population is of 40–50 individuals living in Central Italy, concentrated in the Abruzzo Lazio and Molise National Park [16]. Conservation issues of Marsican brown bear are mainly due to habitat reduction and fragmentation [17, 18]. Furthermore, despite current protective legislation, illegal killings by poaching and poisoning, along with vehicle collisions, represent the main causes of mortality in this species [19]. In recent years, growing attention is paid to the potential role of various pathogens in the health status of the Marsican bear population. However, mortality attributed to infectious diseases in free-ranging brown bears seems to be an overall rare occurrence [20].

In bears, Mycobacteria have been seldom isolated. *M. avium paratuberculosis* was reported as cause of fatal disease in two brown bears (*Ursus arctos*) in Slovakia [21], and an atypical mycobacterium, *M. fortuitum*, was isolated from a brown bear bite wound in a man in Finland [22]. *M. bovis* was only cultured from pooled lymph nodes of a black bear (*Ursus americanus*) but gross or histological lesions were not present [2].

The diagnosis of tuberculosis in free-ranging wildlife relies on post-mortem examination along with histopathology and microbiology [23]. In the case herein presented, the bear was diagnosed with a severe systemic tuberculosis caused by *M. bovis* infection, with both pathological and microbiological aspects suggesting ongoing generalization. A presumptive diagnosis of tuberculosis was initially made based on gross findings, especially on the enlarged necrotic mesenteric lymph nodes and rectal necrotic plaques. Grossly, fibrotic capsules around necrotic tissue or firm nodules on organs surface were lacking in the examined organs. Histopathology confirmed the tubercular nature of infection, showing in every sampled organ specific granulomatous lesion with poorly organized features, along with the presence of acid-fast bacilli, both in the macrophages and extracellularly. The ultimate cause of death of this bear has to be attributed to *M. bovis*, with possible concurrent opportunistic infection with *S. schleiferi* subsp. *coagulans*, known to be associated with Caniformia (Canoidea) hosts [24, 25].

Gross lesions of *M. bovis* infection in cattle are typically caseous and mineralised with histology showing central necrosis surrounded by granulomatous reaction and fibrosis, but lesions in wildlife may differ [23]. Since tuberculosis is a slowly progressive disease, *M. bovis* have been isolated from pooled lymph nodes or other organs in wild carnivores even in the absence of lesions [2, 26, 27]. The case herein described presented with poorly organised lesions, similarly to what have been observed in other carnivores [7, 8, 28–30]. Considering the presence of lesions in multiple organs with different severity stages (ranging from hyperaemia to extensive caseous necrosis), we hypothesized that the mycobacterial infection had a slow course. Late generalisation of the infection via haematogenous spread and concurrent opportunistic infection with Gram-positive bacteria probably intervened in a short span of time, thus justifying the fair body condition of the bear.

Based on massive necrosis of mesenteric lymph nodes and intestinal walls, tuberculosis was thought to be acquired by ingestion. As for the tracing-back of the source of exposure and infection, it was ascertained that *Mycobacterium bovis*-infected cattle had been grazing in the home range of this bear since 2012. Some infected bovines died on pastures and were consumed by scavengers. The epidemiological data were in agreement with the molecular data: the *M. bovis* isolates from the bear and from the cattle herd, when submitted to further molecular characterization, were found indistinguishable both in the spoligotype and in the 12 markers VNTR profile. At present, this genotype does not seem prevalent in Italy. In the database (2008–2018) of the National Reference Laboratory for Bovine Tuberculosis (Istituto Zooprofilattico Sperimentale della Lombardia ed. Emilia-Romagna) with more than 3800 isolates of *M. bovis*/*M. caprae* from around 2260 outbreaks/cases of bovine tuberculosis, this genotype was found only in 6 bovine farms (1 in Abruzzo, 1 in Latium, 1 in Campania, 2 in Apulia, 1 in Calabria), in 15 farms of water buffalo (mainly from Campania) and in one pigs farm in Sicily. Spillover from cattle to bears may have serious implications for the conservation of some relic populations or subspecies of the brown bear, especially in Western Europe.

Eradication campaigns have been conducted in Italy in domestic cattle for decades according to EU and national legislation, thus the overall prevalence of bovine tuberculosis is very low. Nevertheless, in some areas of the Country, cattle population is not tuberculosis-free. In the Abruzzo, Lazio and Molise National Park, traditional farming practices are in place that can affect the risk of transmission of pathogens between domestic cattle and wildlife. Firstly, free-roaming cattle coexists on pastures with both wild ungulates and carnivores. Trades and the practice of summer translocation of cattle to mountain pastures should be fully considered among risk factors for the introduction of bovine tuberculosis in protected areas, similarly to what have been assessed for cattle breakdowns in Northern Italy [31]. Moreover, supplementary feeding points for domestic ungulates in the Park, where carrots or apples are fed to the animals, represent food sources shared between domestic ungulates and wildlife species, posing additional risk of pathogens spillover or spillback [32, 33].

The prevalence of tuberculosis in free-ranging animals has been reported to be related to cases of tuberculosis in domestic cattle [23]. Wild species, especially ungulates, are susceptible to *M. bovis* tuberculosis and could act as reservoirs. Carnivore populations are rather considered spillover hosts that become incidentally infected, therefore they are unlikely to maintain the disease without a continued source of re-exposure, e. g. by preys or scavenged carcasses [34]. Exceptions exist, like some badger populations in England and Ireland, in which the ecology, the social behaviour and densities of this species and its interactions with cattle herds, although still debated, have been deemed to play an important role in the maintenance of infection [35]. In this respect, horizontal (bear-to-bear) transmission of *M. bovis* seems unlikely because routes of horizontal transmission usually include close contact activities such as den sharing, playing, fighting or mating [8], activities that are infrequent in bears as an elusive and solitary species with a low population density.

The case herein reported is the first case of lethal *M. bovis* infection and systemic disease in a free-ranging brown bear. Tuberculosis from *M. bovis* does not pose a serious threat to most wild carnivore populations, but this pathogen could have serious adverse effects in a small and fragmented population such being the case of the Marsican brown bear.

Since *M. bovis* is a well-known zoonotic pathogen and may not be an infrequent finding in wildlife, personal protection equipment when dealing with such forensic cases should always be used as part of biosafety good practices.

Stricter application of health regulations in force is warranted to control the presence of bovine tuberculosis in free-ranging cattle in protected areas. Surveillance of *M. bovis* infections in wild ungulates in the Park, along with wildlife monitoring to assess occurrence of infection in carnivore scavengers, needs to be enhanced to allow a more accurate assessment of the problem. Supplementary feeding points intended for domestic or wild animals should be discontinued in National Parks or protected areas where wildlife and domestic animals share the same pastures, to decrease the risk of pathogens transmission between livestock and wildlife.

## Abbreviations

CO_2_: Carbon dioxide
GC-MS: Gas Chromatography Mass Spectrometry
HE: Hematoxylin-Eosin
IUCN: International Union for Conservation of Nature
PCR: Polymerase Chain Reaction
